# A Transcriptomic Approach Reveals Selective Ribosomal Remodelling in the Tumour Versus the Stromal Compartment of Metastatic Colorectal Cancer

**DOI:** 10.3390/cancers13164188

**Published:** 2021-08-20

**Authors:** Elena Lastraioli, Federico Alessandro Ruffinatti, Francesco Di Costanzo, Cesare Sala, Luca Munaron, Annarosa Arcangeli

**Affiliations:** 1 Department of Experimental and Clinical Medicine, University of Florence, Viale GB Morgagni 50, 50134 Florence, Italy; elena.lastraioli@unifi.it (E.L.); cesare.sala@unifi.it (C.S.); 2 Department of Life Sciences and Systems Biology, University of Torino, Via Accademia Albertina 13, 10123 Torino, Italy; federicoalessandro.ruffinatti@unito.it (F.A.R.); luca.munaron@unito.it (L.M.); 3 Medical Oncology Unit, Azienda Ospedaliero-Universitaria Careggi, Largo Brambilla 3, 50134 Florence, Italy; dicostanzofrancesco@tiscali.it; 4 Complex Dynamics Study Centre (CSDC), University of Florence, 50100 Florence, Italy

**Keywords:** colorectal cancer, transcriptomics, ribosomes, stroma, microdissection

## Abstract

**Simple Summary:**

In this study, we analyzed a cohort of six colorectal cancer patients harboring KRAS mutations and with wild-type BRAF from a transcriptional perspective, with the aim of elucidating the role of the stromal cells in tumor progression. Specifically, paraffin-embedded specimens were subjected to microdissection and hybridized on Agilent-026652 microarrays to compare the gene expression of tumor samples composed of neoplastic epithelial samples against the neighboring stromal tissue. A paired rank-product test led to the detection of 193 differentially expressed genes. Subsequent functional enrichment analysis pointed to extracellular matrix constituents, angiogenesis, and cell migration as the main biological processes enhanced in stromata, while the tumor compartment was characterized by an overexpression of many ribosomal protein genes. A further gene set enrichment analysis against a comprehensive ribosomal protein gene set finally revealed that only cytosolic ribosomes (80S) were affected by such upregulation, while mitochondrial ribosomes were virtually unaltered.

**Abstract:**

Because of its high incidence and poor prognosis, colorectal cancer (CRC) represents an important health issue in several countries. As with other carcinomas, the so-called tumour microenvironment (TME) has been shown to play key roles in CRC progression and related therapeutical outcomes, even though a deeper understanding of the underlying molecular mechanisms is needed to devise new treatment strategies. For some years now, omics technologies and consolidated bioinformatics pipelines have allowed scientists to access large amounts of biologically relevant information, even when starting from small tissue samples; thus, in order to shed new light upon the role of the TME in CRC, we compared the gene expression profiles of 6 independent tumour tissues (all progressed towards metastatic disease) to the expression profile of the surrounding stromata. To do this, paraffin-embedded whole tissues were first microdissected to obtain samples enriched with tumour and stromal cells, respectively. Afterwards, RNA was extracted and analysed using a microarray-based approach. A thorough bioinformatics analysis was then carried out to identify transcripts differentially expressed between the two groups and possibly enriched functional terms. Overall, 193 genes were found to be significantly downregulated in tumours compared to the paired stromata. The functional analysis of the downregulated gene list revealed three principal macro areas of interest: the extracellular matrix, cell migration, and angiogenesis. Conversely, among the upregulated genes, the main alterations detected by the functional annotation were related to the ribosomal proteins (rProteins) of both the large (60S) and small (40S) subunits of the cytosolic ribosomes. Subsequent gene set enrichment analysis (GSEA) confirmed the massive overexpression of most cytosolic—but not mitochondrial—ribosome rProteins.

## 1. Introduction

Solid tumours are not only composed of neoplastic cells but also of the stromal components surrounding the tumour mass and interacting with it—the so-called tumour microenvironment (TME). The TME is a complex network of cells and an extracellular matrix, which is physically part of the tumour itself and can play a central role in tumour progression, both helping neoplastic cells to survive and proliferate in hostile conditions and dictating therapy resistance [1,2,3].

The cellular component of the TME is represented by mesenchymal cells, such as myofibroblasts or specialised cells (e.g., the stellate cells in pancreatic ductal adenocarcinoma) and tumour-associated fibroblasts. In addition, endothelial and muscle cells, as well as cells of both native and adaptive immune systems, contribute to make the TME a highly heterogenous tissue (reviewed in [4]). In more detail, the TME is populated by infiltrating cells such as cancer-associated fibroblasts (CAFs), tumor-associated macrophages (TAMs), endothelial progenitor cells (EPs), mast cells (MCs), mesenchymal stem cells (MSCs), T lympohocyte (CD4, CD8, CD4, Tregs), and platelets [5]. The interactions between the TME and tumour cells must be taken into account, not only because they may affect tumour cell behaviour, but also to derive prognostic information for certain cancers. In particular, the evaluation of stromal gene expression appears particularly relevant [6,7] because the presence of a stroma could affect the proper interpretation of transcriptomic data, leading to mistakes in conclusion drawing [8]. This can be particularly relevant in colorectal cancer (CRC), one of the cancer types with higher incidence and mortality [9], where much effort has been put into in deciphering the molecular characteristics of the cancer tissue in order to better define diagnostic and prognostic biomarkers and identify patient-tailored therapeutic strategies. The relevance of tumour–stroma interactions in CRC was recently reviewed [10], pointing out that stromal compartment also affects clinical features and outcomes. Indeed, tumours with a high amount of stroma are characterised by poorer prognosis [11,12], a fact that was traced back to increased activation of those stroma-induced signals, which ultimately produce an increased tumour aggressiveness. In this line, the so-called tumour/stroma ratio (TSR) [13] was proposed to be included in standard histopathological routines to better classify CRC along with TNM staging [14,15]. In addition to the histopathological characteristics of the stromal component in CRC, the gene expression profiles of tumours and the stromata in CRC merit great attention. In this scenario, the technological acquisition of omics platforms offers the opportunity to collect a huge amount of data from small tissue samples. This approach can, therefore, be applied to tissue biopsies when the starting material is not abundant. Indeed, starting from Sugiyama’s paper [16] using a human cancer pathway finder gene array, several genes involved in angiogenesis, invasion, cell cycle regulation, and proliferation turned out to be differentially expressed in tumour cells compared to healthy colonic epithelium cells and in tumour stromata with respect to normal stromata in CRC patients belonging to different TNM stages [16]. Similarly, a number of differentially expressed genes (for example *HOX2D* and *RHOB*, involved in apoptosis; *SQSTM1*, which mediates NFkB activation; *RRAD*, a member of the Ras/GTPase superfamily) were identified in tumour compared to stromal cells, which were separated using fluorescence-activated cell sorting [17]. Through an RNASeq approach, a transcriptomic analysis was carried out comparing the expression profiles of CRC and healthy colonic mucosa cells, and it was shown that several processes (cell proliferation, inflammatory response, immune response, collagen catabolic process, chemokine-mediated signalling pathway, response to IFNγ) were deregulated [18]. More recently, microRNA (miRNA) expression profiling using a microarray approach revealed the presence of 26 differentially expressed miRNAs involved in cancer development and progression in the tumour versus stromal component of CRC [19]. Overall, despite recent efforts, a clear differential gene expression profile for stromata with respect to tumour cells has not yet been fully elucidated with omics approaches in homogeneous cohorts of CRC patients.

The aim of this study is to compare the transcriptomic profiles of microdissected tumour tissue and the surrounding tumour stromata in metastatic CRC patients harbouring *KRAS* mutations and with wild-type *BRAF* (all progressed towards metastatic disease) through a paired sample design carried out using microarray analysis.

## 2. Materials and Methods

Patients: Ten patients (6 females, 4 males, mean age of 69.7 years, range 60–84) suffering from metastatic colorectal cancer harbouring *KRAS* mutations and not *BRAF*-mutated were enrolled for the study between April 2016 and October 2018 at the Medical Oncology Unit, Azienda Ospedaliero-Universitaria Careggi (Florence). All patients provided informed written consent and the study was approved by the local Ethical Committee of Azienda Ospedaliero-Universitaria Careggi (BIO.16.028, released on 5 October 2016). Paraffin-embedded samples of the primary tumours were retrieved from the archives of the Department of Experimental and Clinical Medicine (University of Florence). The clinical and pathological features of the patients were defined by experienced medical oncologists and pathologists according to the relevant guidelines. Overall, 6 patients were included in the study, since 4 out of 10 were not analysed due to array hybridisation failure (patient characteristics are reported in Table 1).

### 2.1. Sample Preparation

In order to compare tumour tissue with the surrounding stroma, paraffin-embedded samples were manually microdissected. Briefly, 20 μm thick sections were put onto uncharged glass slides and counterstained with haematoxylin. Using a syringe needle, the tumour and stromal tissues were separated under a light microscope, collected, and transferred to a sterile tube.

### 2.2. RNA Extraction and Quality Control

From the enriched samples, total RNA extraction was carried out with the AllPrep^®^ DNA/RNA FFPE kit (Qiagen, Germantown, MD, USA) from all paraffinized tissues for transcriptomics analyses. The RNA concentration was evaluated using a Nanodrop ND-1000 instrument (Thermo Scientific, Waltham, MA USA), while the quality and integrity were evaluated using an Agilent 2100 Bioanalyzer with an RNA 6000 Nano kit (Agilent Technologies, Santa Clara, CA, USA).

### 2.3. Microarray Analysis

Gene expression analysis of RNA was carried out through a One-Color Microarray-Based Gene Expression Analysis using the Agilent-026652 Whole Human Genome Microarray 4 × 44 K v2 platform (Agilent Technologies) and following the manufacturer’s protocols. An Agilent G49000 DA SureScan Microarray scanner was used to scan the microarrays. Data were then extracted by Agilent Feature Extraction and stored for further analyses.

### 2.4. Data Analysis

After array scanning, raw data were processed using a custom script based on R/Bioconductor packages. Specifically, fluorescence intensity signals were background-subtracted using the *normexp* algorithm, log_2_-transformed, and inter-array-normalized through the *quantile–quantile* procedure. Based on the average value of the Agilent Negative Control probes (namely (−)3xSLv1), the gene expression matrix was then subjected to filtering in order to discard unexpressed genes. In particular, only probes featuring log_2_ expression above 6.8 in at least 75% of the samples of at least one group were retained for subsequent analysis. Overall, 25,605 probes out of 34,128 (75.03%) survived the filtering procedure. Upon sample pairing, expression values were used for log_2_ fold change (FC) computation and differential expression assessment according to their rank product statistics (RankProd v3.14.0 Bioconductor package, paired-sample design) [20,21,22,23,24]. *p*-values were adjusted for multiple comparisons and statistical significance was assessed using the Benjamini–Hochberg procedure to control the false discovery rate (BH-FDR) to 5% (i.e., all genes with a *q*-value < 0.05 were considered to be differentially expressed) [25]. Moreover, an additional requirement for FCs was made a posteriori, expunging from DEG lists all genes with |log_2_FC| < 0.5.

The ToppFun web tool (by ToppGene Suite https://toppgene.cchmc.org/, accessed on 12 March 2021) was used for hypergeometric test-based functional enrichment analysis [26]. All terms with a BH-FDR *q*-value < 0.05 were considered statistically significant.

To evaluate the global transcriptional alterations involving ribosomal protein, a comprehensive list of rProtein gene symbols was compiled by sourcing gene symbols from the HGNC database, then a gene set enrichment analysis was performed using GSEA v4.1.0 [27]. To cope with the paired design of our experiment, a preranked list of genes was supplied to the software. Specifically, the *t*-value computed through a preliminary gene-wise paired *t*-test was used as the metric to rank all probes featured in the array. Probes were then collapsed to unique gene symbols before the analysis and a classic (unweighted) enrichment statistic was chosen. Normalized enriched score (NES) and BH-FDR *q*-values are reported in the text for each gene set of interest.

## 3. Results

### 3.1. RNA Extraction and Array Hybridisation

To obtain a comparative transcriptomic profile of both the tumour (i.e., epithelial neoplastic cells) and stromal tissues (i.e., “normal” fibroblasts, endothelial and muscle cells, native and adaptive immune cells) belonging to the same lesion, we performed manual microdissection (Figure S1 and Materials and Methods for details) of paraffin-embedded surgical samples of colorectal cancer patients, with all harbouring *KRAS* mutations and none carrying *BRAF* mutations (whose clinicopathological characteristics are shown in Table 1). Samples enriched in the two components were processed for RNA extraction and then hybridized on Agilent chips (see Supplementary Materials for further details).

### 3.2. Differential Gene Expression

Data obtained from the microarray experiments were preprocessed and analysed according to the informatics pipeline described in the Data Analysis subsection of the Materials and Methods. After a preliminary quality check, only 6 out of 10 samples were retained, since 4 specimens did not meet the required quality standards or failed to hybridise on the chip.

A first step of descriptive statistics was carried out to assess the average inter-patient variability in gene expression for both tissue types considered in this study. Interestingly, the mean inter-patient variance of the stromal compartment turned out to be virtually identical to that of the tumour tissue, although the former typically consists of mixed cell types. In other words, the heterogeneity in stroma composition appeared to be consistent across patients.

**Figure 1:**
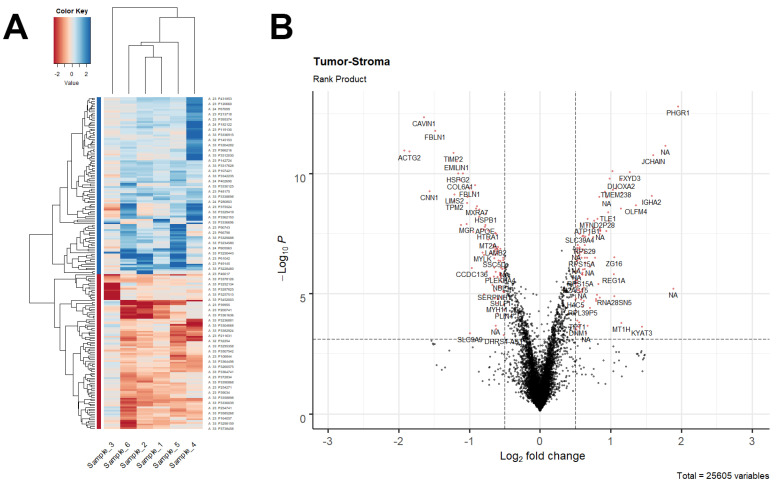
Differential gene expression results from the paired comparison between tumour and stromal samples. (**A**) For each patient (i.e., sample_1 to sample_6), the log_2_FC values of the 193 significant DEGs were heatmapped according to the red-blue colour scale shown in the upper-left corner. Positive log_2_FC values (blue hue) indicate overexpression in tumour compared to stromal samples. Both genes and samples were reordered using a distance-matrix-based hierarchical clustering approach (Euclidean measure and complete linkage), as shown by the dendrograms. (**B**) Volcano plot representing for each probe of the array the negative log_10_(*p*-value) (as returned by the paired rank product test) plotted against the average log_2_FC. Horizontal dashed line is the significance threshold corresponding to a BH-FDR of 5%. Vertical dashed lines are the cut-offs on FCs (|log_2_FC| > 0.5). Red dots represent the 193 DEGs ultimately retained as significant, as reported in Supplementary Table S1.

Afterwards, the two tissue types were statistically tested for differential gene expression using a paired-sample design. Collectively, 193 genes turned out to be significantly deregulated in tumour samples compared to the associated stroma. The top 50 differentially expressed genes (DEGs) sorted by average log_2_ fold change (log_2_FC) are shown in Table 2, while the full DEG set—showing the log_2_FC values observed in each patient—is represented as a heatmap in Figure 1A. In addition, the complete list of DEGs can be found in the Supplementary Materials (Supplementary Table S1). Overall, as shown by the volcano plot in Figure 1B, up- and downregulated genes were well balanced in terms of both probe number (103 upward vs. 90 downward DEGs) and FCs.

### 3.3. Functional Enrichment Analysis

The two lists of statistically significant up- and downregulated genes were separately fed to the ToppFun web tool (https://toppgene.cchmc.org/, accessed on 15 April 2021) for functional enrichment analysis. To this end, ToppFun sources annotations from many different databases, including Gene Ontology (GO), KEGG, BioCarta, Reactome, and HUGO Gene Nomenclature Committee (HGNC). The full lists of the statistically significant terms (*q*-value < 0.05) resulting from the enrichment analysis of both up- and downregulated DEGs are provided as supplementary materials (see Supplementary Tables S2 and S3, respectively).

**Figure 2:**
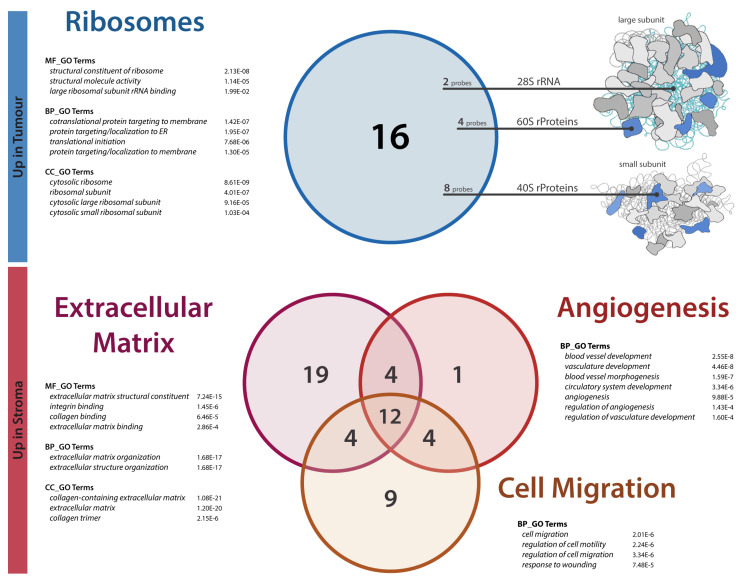
Macro clusters from the functional enrichment analysis of the DEG lists. Upper (blue) section: Cytosolic ribosome structural constituents, translation, and protein targeting to the membrane and ER were the main GO terms that were significantly enriched from the functional analysis of the genes overexpressed in tumours compared to stromata. Except for the two pseudogene probes, all elements of this set are also represented as coloured elements in the ribosome model on the right (modified from KEGG Pathway: Ribosome, 03010 11/12/20, Kanehisa Laboratories). Lower (red) section: Overall, extracellular matrix, angiogenesis, and cell migration GO clusters encompass most of the genes significantly less expressed in tumour than in stromal samples, or equivalently enhanced in the stromal compared to the tumour compartment. In both sections, Venn diagrams show the number of significant probes within each cluster. Below cluster names, the most representative GO terms are reported, together with their q-values as returned from the ToppFun hypergeometric test (MF = molecular function; BP = biological process; CC = cellular component).

The main alterations revealed by the functional annotation of the up-DEG list were related to ribosomal proteins (rProteins), ribosome structural constituents, and protein targeting. In particular, 11 different rProtein gene transcripts—corresponding to 14 unique Agilent probes—related to both the large (60S) and the small (40S) cytosolic ribosome subunits shown to be significantly overexpressed in tumour samples compared to the adjacent stroma (see Table 3 and Figure 2, blue section). Conversely, no genes encoding for rProtein were found within the down-DEG list. In addition, even the product of the *RNA28SN5* gene—the ribosomal RNA giving rise to the 28S subunit—was found to be overexpressed in tumour samples by two independent probes (A_33_P3244165 and A_33_P3336632), further confirming the relevance and the consistency of these data.

On the other hand, from the functional enrichment analysis of the down-DEG list, three main clusters of functional terms emerged: (i) core extracellular matrix (ECM) structure and organization; (ii) angiogenesis, blood vessel development, and morphogenesis; (iii) cell migration and motility (see Figure 2, red section). In more detail, 39 genes could be assigned to the ECM cluster, among which were many collagens (*COL4A2, COL5A1, COL6A1, COL18A1, COL1A1*), ECM glycoproteins (*FBLN1, SPARC, LAMB2, TNXB, EMILIN1, MFAP4*), proteoglycans (*HSPG2, DCN*), and various integrin-related genes (*ITGA5, ITGA7, LIMS2, THY1, TIMP2, TYROBP*). The angiogenesis cluster—besides containing some of the same previous ECM proteins—comprised the angiopoietin *ANGPTL4* and other genes that are known to act in tumour-induced angiogenesis (*AQP1, RAMP1, HSPB1, MYLK*), for a total of 21 entries. Finally, the set related to cell migration accounted for 29 genes, some of which (such as *ARHGAP4*) specifically related to cell motility or migration. For the precise gene content of each cluster, see Supplementary Table S4. Overall, these three functional clusters turned out to encompass the vast majority (67%) of the downregulated genes of our DEG list, albeit with consistent overlap (see Figure 2). Note that since all these three functional groups resulted from the analysis of the down-DEG list only, they should be considered as terms underrepresented in tumour tissue compared to the stromal tissue or overrepresented in the stromal compartment compared to the tumour compartment. In addition to the somewhat expected ECM term, the presence of both the angiogenesis and cell migration clusters could be of particular interest, suggesting a potential pivotal role of the stromata in cancer support and progression.

### 3.4. Ribosomal Gene Set Enrichment Analysis

Because of the remarkable number of ribosomal genes detected when comparing tumour with stroma gene expression, we felt that the global rProteins transcriptional pattern deserved a more thorough investigation. To this end, we sourced gene symbols from the HGNC database to compile a comprehensive list of the human genes coding for cytosolic or mitochondrial rProteins (Supplementary Table S5). We called this list the ribosomal protein gene set (RPGS) and we used it for the gene set enrichment analysis (GSEA) of our transcriptomic dataset to distinguish the different involvement levels of the various ribosomal components in the pathology (see Materials and Methods, Data Analysis subsection). While the previous ToppFun-style enrichment analysis was based on the hypergeometric test of a reduced set of genes (i.e., 193 DEGs), GSEA works with the whole transcriptome—regardless of individual gene statistical significance—allowing the user to evaluate the cumulative contribution of all genes in a given gene set, such as the RPGS [27].

**Figure 3:**
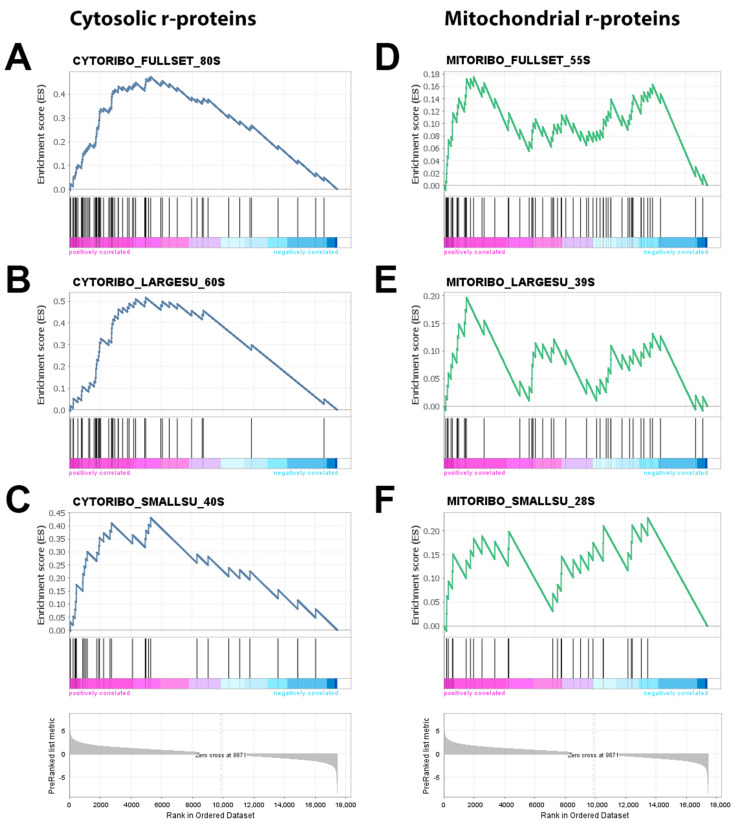
Gene set enrichment analysis of the ribosomal protein gene set. For each gene, a paired *t*-test was computed and the resulting t-value was used as a ranking metric (bottom grey plots). Black bars over the magenta-cyan scales in each subplot indicate the position of each RPGS gene along the ranked gene list. (**A**–**C**) Cytosolic rProtein transcripts were significantly enriched (*q*-value < 10^−3^), whereby genes tended to gather consistently towards the positive edge of the ranked gene list. (**D**,**E**) In contrast, mitochondrial rProtein genes were much more evenly distributed across the ordered dataset.

As expected, GSEA confirmed the strong deregulation of cytosolic (80S) ribosomes, with 58 out of 75 rProtein genes belonging the positive leading edge subset (NES = 4.84, *q*-value < 10^−3^, classic enrichment statistic, Figure 3A), although no relevant differences in rProtein gene overexpression were found between 40S and 60S subunits (Figure 3B,C and Table 4).

Interestingly, the analysis of the mitochondrial rProtein genes resulted in a completely different scenario, since neither the transcripts related to the large subunit (39S) nor those related to the small one (28S) showed any significant enrichment (Figure 3D,F and Table 4).

Based on these observations, GSEA allowed us to conclude that the increased transcription of ribosomal genes in colorectal cancer cells compared to stromal cells mainly relates to the 80S ribosomes, while the mitochondrial ribosomes are left substantially unaffected.

## 4. Discussion

In recent years, several pieces of evidence have pointed out the relevance of the cross-talk between the tumour and the associated stromal components in determining the biological features of a cancer and its progression. Furthermore, omics technologies applied to the analysis of cancer tissues uncovered the possibility that a different molecular profile of differentially expressed genes characterises the tumour tissue and the surrounding stromata. In the present paper, we analysed the gene expression profiles of microdissected tumour and stromal tissues obtained from CRC samples. To avoid conflicting results due to the different clinicopathological characteristics of the patients, we studied a homogeneous cohort of CRC patients, all carrying *KRAS* mutations and none with *BRAF* mutations. We provided evidence that: (i) RNA extracted from enriched tumour and stromal samples, obtained by manual microdissection, can be efficiently used for transcriptomic analysis; (ii) the comparison of the gene expression profiles of tumour and stroma pairs from CRC samples highlighted the presence of several differentially expressed genes (DEGs); (iii) the main overexpressed genes emerging from enriched stroma samples compared to tumour samples belonged to angiogenesis, cell migration, and extracellular matrix functions; (iv) the main result of our analysis is that transcripts related to ribosomes underwent a general overexpression in tumour tissue with respect to stromata, and this increased transcription of ribosomal genes mainly affects the 80S ribosomes, while the mitochondrial ribosomes are not affected.

From a technical point of view, our results regarding the separation (and cell component enrichment) of tumour tissue vs. stroma tissue are similar to those reported by Carlomagno’s group in terms of showing the efficacy of manual microdissection as a separation technique, although they applied a slightly different protocol to obtain enriched samples from paraffin-embedded specimens to be used for transcriptomic analysis [19]. Our approach for microdissection is simpler and quicker and has the advantage of preserving the original tissue specimen, which can be used for further applications. To our knowledge, the paper by Scarpati and colleagues is one of the few to have applied a similar approach, although it was focused on miRNA analysis, while our contribution is aimed at evaluating the expression profile of the whole transcriptome in CRC cancer and stromal cells.

Overall, in our cohort of 6 metastatic CRC patients all harbouring *KRAS* mutations and none carrying *BRAF* mutations, 193 genes turned out to be significantly deregulated in the tumour compared to the paired stroma. Although we applied a conservative analysis after proper filtering and cleaning of the background noise, the number of DEGs we found was higher than reported in other papers presenting omics data, such as the study by Sugiyama et al., 2005 [16]; however, despite the higher number of DEGs (193 vs. 6) and differences in the methodology used, some of our results agree with those reported by Sugiyama and colleagues. Indeed, the main pathways identified as upregulated in the stromal tissue by Sugiyama and colleagues were the same ones we observed as downregulated in the tumour component when compared to the stromal component, namely angiogenesis, cell migration, and the extracellular matrix; however, caution is warranted when looking at the 39 DEGs belonging to the ECM functional cluster, because they could be—at least in part—a consequence of our particular experimental design (i.e., the direct comparison between the tumour epithelium and the neighbouring stromal compartment), rather than representing a genuine pathological CRC signature. Indeed, since a number of ECM-related genes are known to be constitutively expressed in stromata but very few are found in the tumour epithelium, their apparent downregulation in tumour samples could simply reflect the different compositions of the two tissues being compared. On the other hand, some of the ECM genes we labelled as negatively regulated in the epithelium could actually be upregulated in stromata, taking part in the definition of the tumour microenvironment and the CRC-specific extracellular matrix signature, as suggested in other recent studies [28]. Likewise, both the proangiogenic and the promigratory genes we found to be upregulated in stromata compared to tumours arguably indicate expression of the stromal involvement in terms of cancer growth and metastasis development, even if some of them could also be related to the physiological differences between the two tissue types. Regarding the cell migration functional cluster in particular, it is interesting to observe the presence of some genes taking part in intracellular promigratory signalling pathways (*ARHGAP4, GNAI2, AQP1, HSPB1*) and others being implicated in the secretion of promigratory proteins (*SPARC, NBL1, LGALS1*), possibly acting as a chemoattractant source for the tumour cells in the neighbouring compartment. In this regard, *SPARC* (secreted protein acidic and cysteine-rich) has already been reported in different types of metastatic cancer—including CRC—and has attracted attention as a possible biomarker [29,30].

Different reports have addressed the DEG profile in CRC, although most of them have been focused on comparisons between CRC and healthy colon samples. Nevertheless, the relevance of separately evaluating the two components was pointed out several years ago by Smith and colleagues, who showed that some DE genes were detected when comparing laser-microdissected tumours with the whole lesion [16], although they did not compare tumour vs. stroma tissues, as in the present paper.

The main and more interesting finding of our study is the strong evidence of increased expression of ribosomal genes in tumour CRC tissue with respect to the corresponding stromal tissue in a paired design. To corroborate this fundamental finding, we used UCSC Xena Browser (University of California, Santa Cruz, http://xena.ucsc.edu/, accessed on 5 August 2021) [31] for the direct comparison of tumour expression data stored in the TCGA database with the normal (i.e., non-tumour) samples from GTEx (https://gtexportal.org/home/, accessed on 5 August 2021) [32]. Xena allowed us to explore the differential expression of the 9 rProtein gene transcripts that were significantly deregulated in our study (see Table 2). To make this in silico comparison in our experimental study, we filtered TCGA data in order to keep only colon cancer samples from patients harbouring *KRAS* mutations and with wild-type *BRAF*. This led to a final cohort of *n* = 118 tumour samples and *n* = 304 normal tissues (cohort assembling and filtering procedures are detailed in Supplementary Materials, Section S2). Notably, all rProtein genes we detected as overexpressed in our study were also significantly upregulated when looking at the data deposited in TCGA and GTEx databases (Figures S3 and S4); however, it is worth noting that normal samples from GTEx are not subjected to microdissection, nor any other sub-tissue selection procedure. For this reason, results retrieved by Xena cannot be considered as totally faithful validation of our study, since our experimental design programmatically compared the tumour epithelium with the adjacent stromal tissue, rather they represent a strong indication that rProtein gene upregulation is a real hallmark of colorectal cancer pathology, as well as a distinctive feature of the transformed epithelial tissue compared to the underlying stroma.

A distinctive feature of cancer cells is the constitutive activation of growth factor signalling pathways that alter the activity of transcription factors [33,34] responsible for the increased ribosome biogenesis due to the hyperactivation of RNA polymerases I and III [35,36]. Different reports have highlighted that ribosome biogenesis has a role in the initial phases of CRC tumour progression, mainly due to rRNA polymerase (or its cofactors) overexpression or mutation [37,38]. Another study [39] demonstrated that a germline mutation in *RPS20,* encoding a component of the small ribosomal subunit, increases the predisposition to develop a particular form of hereditary CRC named familial colorectal cancer type X (FCCX).

In general, the remodelling of the ribosomal machinery is likely to exert relevant biological effects in terms of protein synthesis and targeting, and could be integrated with the investigation of the translational and post-translational modifications in CRC. It is generally accepted that tumour cells, being characterised by enhanced growth, might also have increased ribosome production associated with altered biogenesis and nucleolar modifications (in either number, shape, or size) [37]. Moreover, the mounting evidence highlights the strong links between rRNA synthesis and rProteins with the development of human cancers [38].

Overall, our data highlight the relevance of 80S ribosomes in cancer, which are in line with the recently proposed use of these organelles for innovative therapies (reviewed in [40]).

Since all of the patients carried *KRAS* mutations, our results are in accordance with a recent report [41] showing that CRC cell lines harbouring *KRAS* mutations in codon 13 are characterised by a significant upregulation of several genes involved in ribosome genesis, metabolism, and mRNA translation paralleled by increased proliferation and protein synthesis.

Another interesting finding obtained in this study was the observation that the affected ribosomes belong to the cytosolic compartment, while mitochondrial ribosomes are not significantly altered. The link between cancer and metabolism was discovered many years ago, thanks to pivotal studies on aerobic glycolysis in cancer [42], and has experienced a great resurgence in recent years (reviewed in [43]).

The Warburg effect was shown to be a finely regulated metabolic state that can represent an advantage for cancer cells in the context of increased biosynthetic demand [44]. It is well known that at least some types of cancer cells have increased glycolysis that might be paralleled by a downregulation of oxidative phosphorylation (OXPHOS) [45].

The mitochondrial remodelling in *KRAS* mutated–*BRAF* wild-type CRC agree with Warburg’s hypothesis, suggesting that the scenario might be different to that observed in other tumours, as in the case of the relevant role of OXPHOS in lymphomas [46].

It is interesting to notice that the cohort analysed in this paper was composed of surgically resected CRC belonging to TNM stages II–IV, although all of the patients were enrolled when the disease became metastatic; therefore, it could be argued that the molecular signature we described could identify more aggressive cancers that are likely to progress towards a systemic disease.

The data provided in this paper are promising but deserve further investigation. One option for further study could be a comparison between tumour tissue and healthy colorectal mucosa, as well as between a tumour stroma and normal stroma. It would also be useful to extend the analysis to different tumours in order to understand whether this DEG profile is unique to CRC or could represent a more general pattern. Moreover, proteomic screening could provide different and complementary information compared to the transcriptomic analysis presented herein.

## 5. Conclusions

Tumour tissue and the surrounding stroma tissue are characterised by the differential expression of several genes. Omics approaches could help in identifying molecular patterns useful for the management of cancer patients. Data reported in this study represent a first piece of evidence pointing to the overexpression of cytosolic ribosome transcripts in CRC tumours vs. stromata. Further analyses performed in larger cohorts and different neoplasias are warranted.

## Figures and Tables

**Table 1:** Demographic and clinical features of the patients enrolled in the study.

Feature	Distribution within the Cohort
Gender	Female, 5 (83.3%)
	Male, 1 (16.7%)
Localisation	Right colon, 4 (66.6%)
	Transverse, 1 (16.7%)
	Rectum, 1 (16.7%)
Grading	G1, 0 (0%)
	G2, 3 (50.0%)
	G3, 1 (16.7%)
	Undefined, 2 (33.3%)
Mucinous	No, 3 (50.0%)
	Yes, 3 (50.0%)
TNM stage at diagnosis	IIa, 2 (33.3%)
	IIIb, 1 (16.7%)
	IV, 3 (50.0%)
Metastases	Liver, 5
	Lung, 2
	Abdominal Lymph nodes, 3
	Other, 2
Pleural effusion	No, 5 (83.3%)
	Yes, 1 (16.7%)
Ascitic effusion	No, 4 (66.7%)
	Yes, 2 (33.3%)
Local relapse	No, 6 (100%)
	Yes, 0 (0.0%)
Vascular invasion	No, 6 (100.0%)
	Yes, 0 (0.0%)
Perineural invasion	No, 6 (100%)
	Yes, 0 (0.0%)
*KRAS* mutations	No, 0 (0.0%)
	Codon 12, 4 (66.7%)
	Codon 13, 2 (33.3%)
*BRAF* mutations	No, 6 (100.0%)
	Yes, 0 (0.0%)

**Table 2:** Top 50 differentially expressed genes resulting from the statistical comparison of tumour vs. stromal samples (Rank Product, paired design). Positive log_2_FC values indicate overexpression in tumours compared to stromata.

Probe ID	Gene Symbol	Description	log_2_FC	*p*-Value	*q*-Value BH-FDR
A_33_P3323501	PHGR1	proline, histidine, and glycine-rich 1	1.95	1.60 × 10^−13^	4.09 × 10^−9^
A_23_P167168	JCHAIN	joining chain of multimeric IgA and IgM	1.60	1.73 × 10^−11^	1.47 × 10^−7^
A_23_P61042	IGHA2	immunoglobulin heavy constant alpha 2 (A2m marker)	1.58	8.54 × 10^−10^	1.82 × 10^−6^
A_23_P46017	KYAT3	kynurenine aminotransferase 3	1.44	2.38 × 10^−4^	2.12 × 10^−2^
A_24_P181254	OLFM4	olfactomedin 4	1.35	2.06 × 10^−9^	3.51 × 10^−6^
A_33_P3228460	FXYD3	FXYD domain containing ion transport regulator 3	1.27	8.47 × 10^−11^	4.34 × 10^−7^
A_23_P393099	TFF3	trefoil factor 3	1.16	6.16 × 10^−10^	1.43 × 10^−6^
A_33_P3250443	DUOXA2	dual oxidase maturation factor 2	1.15	1.81 × 10^−10^	6.64 × 10^−7^
A_33_P3368313	MT1H	metallothionein 1H	1.15	1.67 × 10^−4^	1.62 × 10^−2^
A_24_P844984	PIGR	polymeric immunoglobulin receptor	1.14	2.86 × 10^−9^	4.58 × 10^−6^
A_33_P3362153	TMEM238	transmembrane protein 238	1.08	4.10 × 10^−10^	1.31 × 10^−6^
A_33_P3244165	RNA28SN5	RNA, 28S ribosomal N5	1.05	1.31 × 10^−5^	2.40 × 10^−3^
A_23_P49145	ZG16	zymogen granule protein 16	1.05	3.06 × 10^−7^	1.57 × 10^−4^
A_23_P90743	REG1A	regenerating family member 1 alpha	1.05	1.55 × 10^−6^	5.23 × 10^−4^
A_23_P208788	C19orf33	chromosome 19 open reading frame 33	1.03	7.92 × 10^−11^	5.07 × 10^−7^
A_33_P3329088	PRSS8	serine protease 8	0.99	1.60 × 10^−10^	6.81 × 10^−7^
A_33_P3257678	H3C15	H3 clustered histone 15	0.97	9.43 × 10^−9^	1.15 × 10^−5^
A_23_P402751	TLE1	TLE family member 1, transcriptional corepressor	0.97	4.15 × 10^−9^	6.25 × 10^−6^
A_23_P95790	ITLN1	intelectin 1	0.94	2.45 × 10^−8^	2.42 × 10^−5^
A_33_P3385006	SLC39A5	solute carrier family 39 member 5	0.92	5.67 × 10^−10^	1.45 × 10^−6^
A_23_P58266	S100P	S100 calcium binding protein P	0.85	2.02 × 10^−8^	2.15 × 10^−5^
A_23_P79562	FABP1	fatty acid binding protein 1	0.84	3.30 × 10^−8^	2.91 × 10^−5^
A_33_P3338698	IHH	Indian hedgehog signaling molecule	0.84	9.19 × 10^−10^	1.81 × 10^−6^
A_23_P76961	RPS29	ribosomal protein S29	0.63	9.72 × 10^−8^	6.91 × 10^−5^
A_33_P3393821	C1R	complement C1r	−0.86	3.16 × 10^−9^	4.04 × 10^−6^
A_23_P121533	SPON2	spondin 2	−0.86	6.11 × 10^−9^	7.11 × 10^−6^
A_33_P3239587	MXRA7	matrix-remodeling-associated 7	−0.89	2.27 × 10^−9^	3.23 × 10^−6^
A_23_P64873	DCN	decorin	−0.91	2.86 × 10^−9^	3.86 × 10^−6^
A_33_P3236858	TGFB1I1	transforming growth factor beta 1 induced transcript 1	−0.92	3.07 × 10^−10^	7.87 × 10^−7^
A_23_P200741	DPT	dermatopontin	−0.94	2.66 × 10^−8^	2.27 × 10^−5^
A_33_P3298159	PTGDS	prostaglandin D2 synthase	−0.95	5.64 × 10^−10^	1.11 × 10^−6^
A_33_P3287825	CCDC136	coiled-coil domain containing 136	−0.96	8.63 × 10^−7^	3.40 × 10^−4^
A_23_P211631	FBLN1	fibulin 1	−0.99	4.03 × 10^−10^	9.37 × 10^−7^
A_32_P46214	SLC9A9	solute carrier family 9 member A9	−0.99	4.47 × 10^−4^	3.40 × 10^−2^
A_23_P205031	COL4A2	collagen type IV alpha 2 chain	−1.02	9.39 × 10^−10^	1.60 × 10^−6^
A_23_P50946	RAMP1	receptor-activity-modifying protein 1	−1.03	1.68 × 10^−9^	2.53 × 10^−6^
A_33_P3361636	MGP	matrix Gla protein	−1.04	1.28 × 10^−8^	1.42 × 10^−5^
A_33_P3330039	PLEKHO1	pleckstrin homology domain-containing O1	−1.09	9.78 × 10^−11^	3.58 × 10^−7^
A_33_P3233125	PSD	pleckstrin and Sec7 domain-containing	−1.11	1.41 × 10^−8^	1.45 × 10^−5^
A_32_P32254	COL6A1	collagen type VI alpha 1 chain	−1.13	1.91 × 10^−10^	5.44 × 10^−7^
A_33_P3321657	HSPG2	heparan sulfate proteoglycan 2	−1.16	9.81 × 10^−11^	3.14 × 10^−7^
A_33_P3333455	EMILIN1	elastin microfibril interfacer 1	−1.18	2.99 × 10^−11^	1.28 × 10^−7^
A_33_P3268304	LIMS2	LIM zinc finger domain-containing 2	−1.21	7.61 × 10^−10^	1.39 × 10^−6^
A_23_P216501	TPM2	tropomyosin 2	−1.21	1.38 × 10^−9^	2.21 × 10^−6^
A_33_P3382177	TIMP2	TIMP metallopeptidase inhibitor 2	−1.23	1.35 × 10^−11^	6.89 × 10^−8^
A_33_P3249872	FBLN1	fibulin 1	−1.48	1.62 × 10^−12^	2.07 × 10^−8^
A_23_P125233	CNN1	calponin 1	−1.56	5.52 × 10^−10^	1.18 × 10^−6^
A_23_P394064	CAVIN1	Caveolae-associated protein 1	−1.64	4.54 × 10^−13^	1.16 × 10^−8^
A_23_P39955	ACTG2	actin gamma 2, smooth muscle	−1.85	1.21 × 10^−11^	7.74 × 10^−8^
A_33_P3275801	DES	desmin	−1.92	1.07 × 10^−11^	9.12 × 10^−8^

**Table 3:** List of the ribosomal protein genes upregulated in the tumour compartment compared to the stromal compartment. For multiple significant probes targeting the same gene (see # probes column), the higher log_2_FCs and the lower *p*-values are reported.

Gene Symbol	Gene Name	log_2_FC	*p*-Value	*q*-Value	# Probes
RPL23A	ribosomal protein L23a	0.54	7.08 × 10^−6^	1.56 × 10^−3^	1
RPL31	ribosomal protein L31	0.58	1.77 × 10^−5^	2.95 × 10^−3^	1
RPL37A	ribosomal protein L37a	0.62	5.67 × 10^−6^	1.37 × 10^−3^	1
RPL39	ribosomal protein L39	0.62	2.02 × 10^−5^	3.28 × 10^−3^	1
RPS7	ribosomal protein S7	0.57	2.14 × 10^−4^	1.98 × 10^−2^	1
RPS15A	ribosomal protein S15a	0.59	3.29 × 10^−7^	1.56 × 10^−4^	2
RPS19	ribosomal protein S19	0.59	1.55 × 10^−6^	5.17 × 10^−4^	1
RPS21	ribosomal protein S21	0.63	1.88 × 10^−7^	1.07 × 10^−4^	2
RPS29	ribosomal protein S29	0.63	9.72 × 10^−8^	6.91 × 10^−5^	2
RNA28SN5	RNA, 28S ribosomal N5	1.05	1.31 × 10^−5^	2.40 × 10^−3^	2
RPL39P5	ribosomal protein L39 pseudogene 5	0.61	3.49 × 10^−5^	5.11 × 10^−3^	1
RPL32P3	ribosomal protein L32 pseudogene 3	0.56	1.61 × 10^−4^	1.59 × 10^−2^	1

**Table 4:** GSEA statistics resulting from the analysis of the RPGS. Classic enrichment statistics and a preranked gene list based on a preliminary paired t-test were used (ES = enrichment score; NES = normalized enrichment score; -- = empty leading edge subset).

Gene Set	Size	ES	NES	Nominal *p*-Value	*q*-Value BH-FDR	Leading Edge
CytoRibo_FullSet_80S	75	0.47	4.84	<10^−3^	<10^−3^	58 (77.3%)
CytoRibo_LargeSU_60S	45	0.52	4.10	<10^−3^	<10^−3^	36 (80.0%)
CytoRibo_SmallSU_40S	30	0.43	2.81	<10^−3^	<10^−3^	22 (73.3%)
MitoRibo_FullSet_55S	66	0.18	1.68	0.029	0.048	19 (28.8%)
MitoRibo_LargeSU_39S	39	0.20	1.45	0.092	0.108	11 (28.2%)
MitoRibo_SmallSU_28S	27	0.23	1.41	0.111	0.107	--

## Data Availability

Data are available upon reasonable request.

## Supplementary Materials

The following are available online at https://www.mdpi.com/article/10.3390/cancers13164188/s1: Figure S1: Sample Preparation, Figure S2: Xena Query, Figure S3: Xena Column View, Figure S4: Xena Chart View, Table S1: Full DEG List, Table S2: Overexpressed Terms, Table S3: Underexpressed Terms, Table S4: Main Cluster Gene List, Table S5: Ribosomal GeneSet.
