# Supplementary material for: A Transcriptomic Approach Reveals Selective Ribosomal Remodelling in the Tumour Versus the Stromal Compartment of Metastatic Colorectal Cancer
Source: Cancers (Basel). 2021 Aug 20;13(16):4188. doi: 10.3390/cancers13164188 (PMC8394399; doi:10.3390/cancers13164188)
Supplement: Supplementary file 1 [file cancers-13-04188-s001.zip › Supplementary Materials.pdf]

cancers

MDPI

Supplementary Materials: A Transcriptomic Approach Reveals Selective Ribosomal Remodelling in the Tumour Versus the Stromal Compartment of Metastatic Colorectal Cancer

Elena Lastraioli, Federico Alessandro Ruffinatti, Francesco di Costanzo, Cesare Sala, Luca Maria Munaron and Annarosa Arcangeli

1. Sample Processing: from RNA Extraction to Array Hybridisation

Paraffin-embedded surgical samples of colorectal cancer patients all harbouring KRAS mutations and none carrying BRAF mutations were subjected to manual microdissection of both the tumour (i.e., epithelial neo-plastic cells) and the stromal tissue (i.e., "normal" fibroblasts, endothelial and muscle cells, native and adaptive immune cells) belonging to the same lesion (Figure S1A). Samples hence enriched in the two components, were processed for RNA extraction, and then hybridized on Agilent Chip. Only samples with a good integrity index of the extracted RNA (a representative sample is reported in Figure S1B) were further processed and submitted to microarray analysis. After hybridisation, the slides were scanned (Figure S1C), then data were extracted aligning the grid to the spots and setting up the correct analysis type (Figure S1D). Figure S1E shows the Spike-In values along with the linear regression statistics, representing an internal quality control of each microarray experiment.

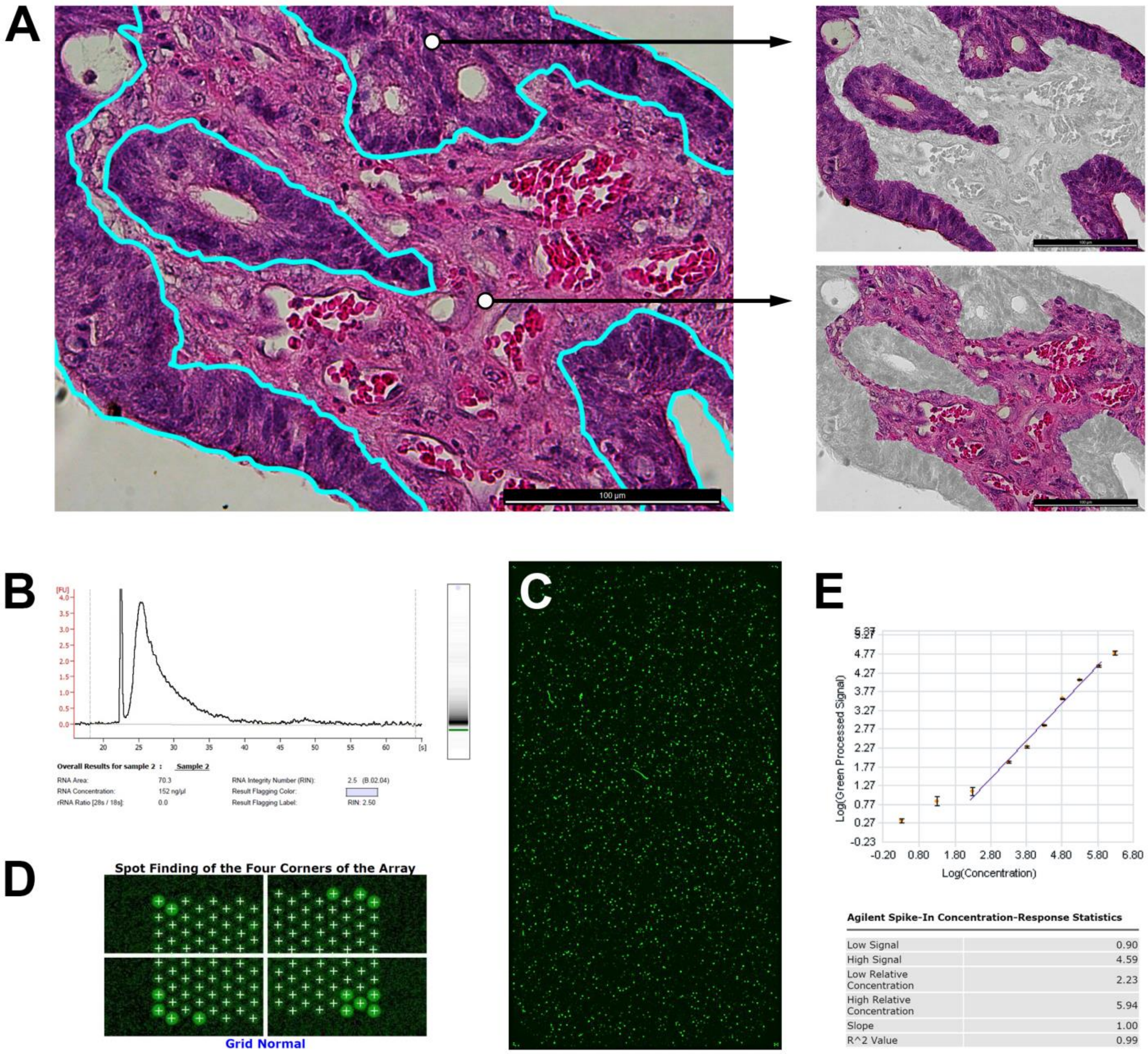

Figure S1. Sample processing, evaluation of RNA quality, and microarray hybridisation. (**A**) Enrichment of tumour and stroma samples through manual microdissection from paraffin-embedded specimens. (**B**) Agilent 2100 Bioanalyzer analysis of a representative specimen showing RNA Integrity Number (RIN = 2.5) consistent with the values generally expected for paraffin-embedded tissues. (**C**) Representative scanning of a microarray slide after hybridisation. (**D**) Magnification of the four corners of the slide in (C) showing reference probes for grid positioning. (**E**) Upper box: log (Signal) vs log (Relative Concentration) plot for the ten (+)E1A_r60 positive control transcripts of the Agilent One-Color RNA Spike-In Kit. Lower Box: statistics for the regression of the fitting line in the linear region. .

2. Validation through TCGA and GTEx Databases

To corroborate our primary finding, as discussed in the main text, we used UCSC Xena Browser (University of California, Santa Cruz, http://xena.ucsc.edu/, accessed on 05 August 2021)[1] to explore the differential expression of the 9 rProtein gene transcripts resulted significantly deregulated in our study (see Table 2 in the main text). Notably, Xena Browser allows the direct comparison of tumour expression data stored in TCGA with the normal (i.e., non-tumour) samples from GTEx (https://gtexportal.org/home/)[2]. Although of different origin, expression data retrieved by Xena are fully comparable since

all samples have been re-analyzed using the same RNA-seq pipeline, thus eliminating the batch effect due to different computational processing. Thanks to this novel web tool, it is possible to overcome the well-known issue concerning the typical lack of normal reference samples in TCGA and ultimately conduct more robust and reliable statistical comparisons. More in detail, we filtered TCGA data in order to keep just colon cancer samples from patients harbouring *KRAS* mutations and with wild type *BRAF*. As for the control group, all the normal-colon samples retrieved from GTEx could be used. This led to a final comparison between n=118 tumour samples and n = 304 normal tissues. Cohort assembling and filtering procedures are depicted in Figure S2, while Figure SM3 shows Xena output containing the heatmap of the differential expression. Notably, all the rProtein genes we detected as overexpressed in our study also resulted to be significantly upregulated when looking at the data deposited in TCGA and GTEx databases (Figure S4).

```
Xena
|
|--- TCGA TARGET GTEx (A)
|
|--- Genomic
|     |--- Gene Expression (B)
|
|--- Phenotypic
|     |--- study (C)
|     |--- sample type (D)
|     |--- primary_site (G)
|
|--- Add Column
|     |--- Genomic --> KRAS --> TCGA somatic mutations (Pan-cancer Atlas MC3 public version) (E)
|     |--- Genomic --> BRAF --> TCGA somatic mutations (Pan-cancer Atlas MC3 public version) (F)
|
|                                                                                             Size:
|--- Filter                                                                                   19131
      |--- [Colon as Primary Site] G:colon --> Keep                                           639
      |--- [Wild Type BRAF] --> F:silent OR F:missense OR F:nonsense --> Remove               594
      |--- [KRAS Mutations] --> (C:GTEX) OR (C:TCGA AND D:primary AND E:missense) --> Keep    430
      |--- [NO KRAS mutations in normals] --> E:silent OR E:3'UTR --> Remove                  426
      |--- [Outlier samples] --> A:5CH --> Remove                                             422
```

Figure S2. Textual-tree representation of our TCGA-GTEx query through Xena. Each capital letter within round brackets denotes a particular factor contributing to the final experimental design, as represented in Figure SM3. In the last branch of the tree, the different filter layers we applied to get the final cohort of n = 422 samples are listed.

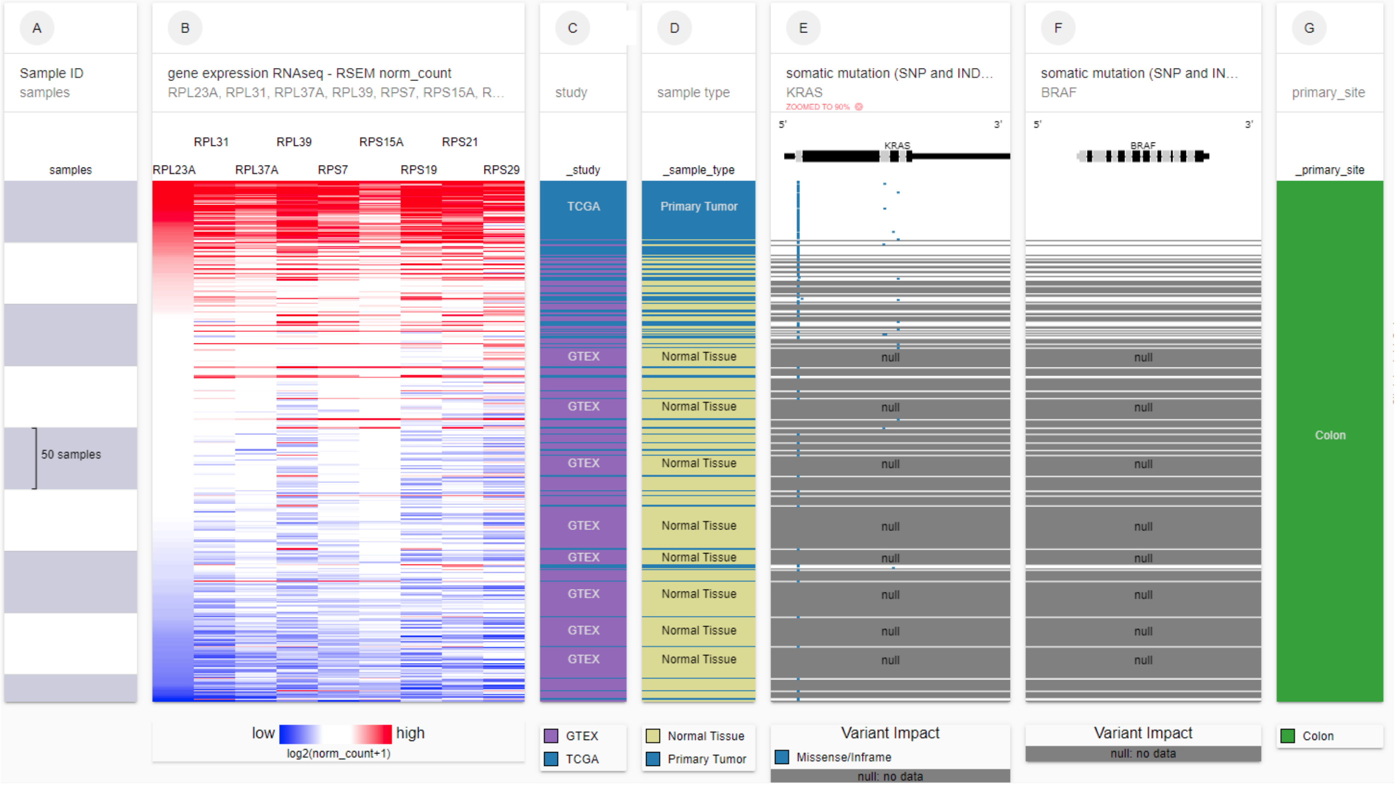

Figure S3. Xena "column view" of the query results. Each column corresponds to one of the factors we selected during cohort assembling. Gene expression levels for the 9 genes of interest (**B**) have been retrieved from a cohort of 422 samples (**A**), consisting of n = 118 tumour samples from TCGA and n = 304 normal tissues from GTEx (**C**,**D**). TCGA samples were filtered in order to have a KRAS-mutated and BRAF-wild type genotype (**E**,**F**, respectively). All samples were of colonic origin (**G**).

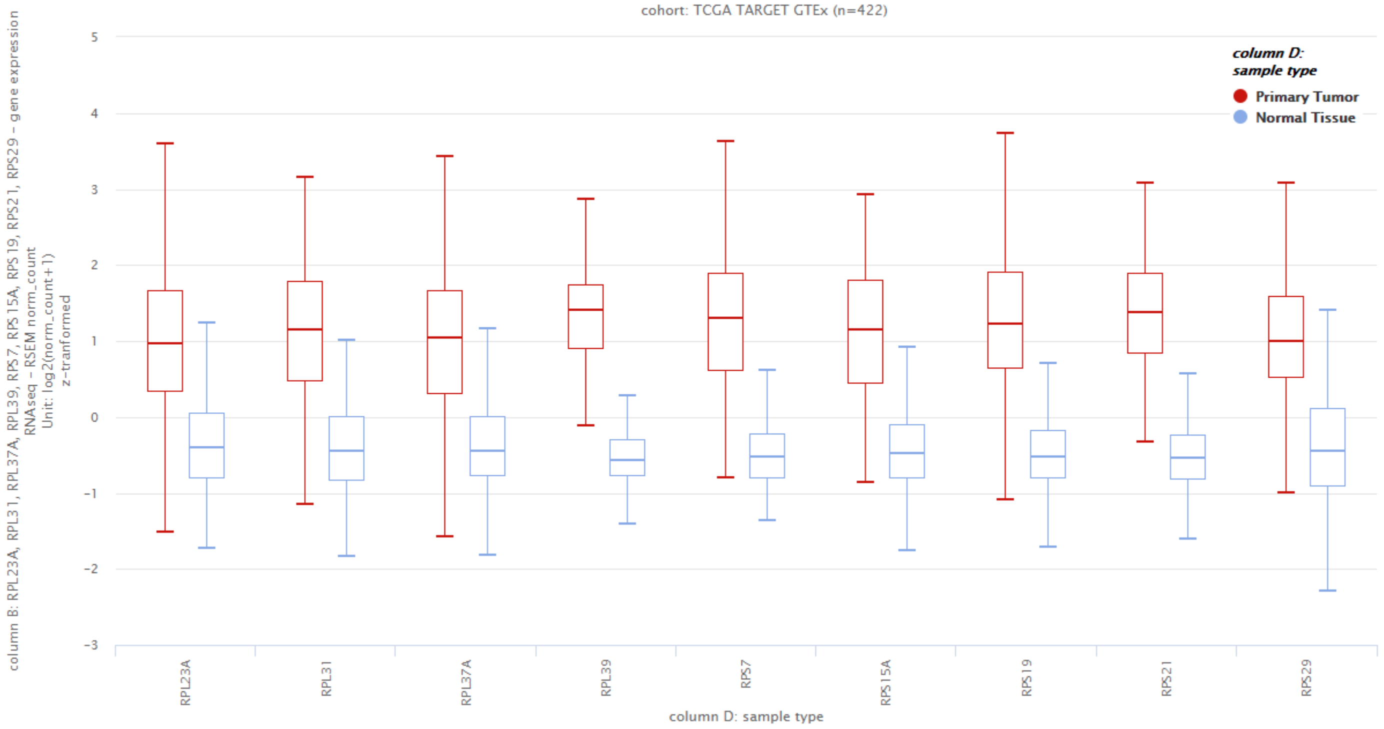

Figure S4. Xena "chart view" of the query results. For each gene of interest, box plots represent the z-scored expression data (Figure S3B) as a function of the explanatory variable "sample type" (Figure S3D). Gene expression is given in terms

of z-scores of the log2(normalized counts + 1). For each rProtein gene a Welch's *t*-test was conducted returning virtually null *p*-values and *t*-statistics ranging between $t = 12.10$ (for RPL23A) and $t = 26.96$ (for RPL39).

Even if these results are in complete agreement with what we found using expression microarrays, it is worth noting that normal samples from GTEx are not subjected to microdissection nor any other sub-tissue selection procedure. As for transverse colon samples in particular, GTEx Tissue Harvesting Work Instruction [3] establishes that *"aliquots should contain the full thickness of the colonic wall, i.e., mucosa and muscularis propria"*. For this reason, results retrieved by Xena cannot be considered a totally faithful validation of our study, since our experimental design programmatically compared tumour epithelium with the adjacent stromal tissue. Rather, they represent a strong indication that rProtein gene upregulation is a genuine hallmark of colorectal cancer pathology, as well as a distinctive feature of the transformed epithelial tissue compared to the underlying stroma.

References

1. Goldman, M.J.; Craft, B.; Hastie, M.; Repečka, K.; McDade, F.; Kamath, A.; Banerjee, A.; Luo, Y.; Rogers, D.; Brooks, A.N.; et al. Visualizing and interpreting cancer genomics data via the Xena platform. *Nat. Biotechnol.* **2020**, *38*, 675–678. doi: 10.1038/s41587-020-0546-8.
2. GTEx Consortium. The GTEx Consortium atlas of genetic regulatory effects across human tissues. Science **2020**, 369, 1318–1330. doi: 10.1126/science.aaz1776.
3. Available online: https://biospecimens.cancer.gov/resources/sops/library.asp (accessed on 05 August 2021).
